# Estimated costs of hospitalization due to coronary artery disease attributable to familial hypercholesterolemia in the Brazilian public health system

**DOI:** 10.20945/2359-3997000000030

**Published:** 2018-05-07

**Authors:** Luciana R. Bahia, Roger S. Rosa, Raul D. Santos, Denizar V. Araujo

**Affiliations:** 1 Universidade do Estado do Rio de Janeiro Universidade do Estado do Rio de Janeiro Instituto de Avaliação de Tecnologia em Saúde Departamento de Medicina Interna Rio de Janeiro RJ Brasil Departamento de Medicina Interna, Universidade do Estado do Rio de Janeiro (UERJ); Instituto de Avaliação de Tecnologia em Saúde (IATS), Rio de Janeiro, RJ, Brasil; 2 Universidade Federal do Rio Grande do Sul Universidade Federal do Rio Grande do Sul Departamento de Medicina Social Porto Alegre RS Brasil Departamento de Medicina Social, Universidade Federal do Rio Grande do Sul (UFRGS), Porto Alegre, RS, Brasil; 3 Universidade de São Paulo Universidade de São Paulo Faculdade de Medicina Instituto do Coração do Hospital das Clínicas, Unidade Clínica de Lípides São Paulo SP Brasil Unidade Clínica de Lípides, Instituto do Coração do Hospital das Clínicas da Faculdade de Medicina da Universidade de São Paulo (InCor/HCFMUSP); Programa de Centro de Medicina Preventiva e Cardiologia, Hospital Albert Einstein (HIAE), São Paulo, SP, Brasil

**Keywords:** Costs and cost analysis, coronary artery disease, familial hypercholesterolemia, Brazil, hospitalization

## Abstract

**Objective:**

Cardiovascular diseases are the leading cause of death in Brazil, imposing substantial economic burden on the health care system. Familial hypercholesterolemia (FH) is known to greatly increase the risk of premature coronary artery disease (CAD). This study aimed to estimate the economic impact of hospitalizations due to CAD attributable to FH in the Brazilian Unified Health Care System (SUS).

**Subjects and methods:**

Retrospective, cross-sectional study of data obtained from the Hospital Information System of the SUS (SIHSUS). We selected all adults (≥ 20 years of age) hospitalized from 2012­-2014 with primary diagnoses related to CAD (ICD-10 I20 to I25). Attributable risk methodology estimated the contribution of FH in the outcomes of interest, using international data for prevalence (0.4% and 0.73%) and relative risk for events (RR = 8.56).

**Results:**

Assuming an international prevalence of FH of 0.4% and 0.73%, of the 245,981 CAD admissions/year in Brazil, approximately 7,249 and 12,915, respectively, would be attributable to an underlying diagnosis ­­of FH. The total cost due to CAD per year, considering both sexes and all adults, was R$ 985,919,064, of which R$ 29,053,500 and R$ 51,764,175, respectively, were estimated to be attributable to FH. The average cost per FH-related CAD event was R$ 4,008.

**Conclusion:**

Based on estimated costs of hospitalization for CAD, we estimated that 2.9-5.3% are directed to FH patients. FH can require early specific therapies to lower risk in families. It is mandatory to determine the prevalence of FH and institute appropriate treatment to minimize the clinical and economic impact of this disease in Brazil.

## INTRODUCTION

Cardiovascular diseases (CVDs) are the main cause of death in Brazil, with coronary artery disease (CAD) being the main cause among all CVDs (31%), followed by cerebrovascular disease (30%), and heart failure (18%). A significant reduction in the mortality associated with CVDs has been demonstrated over the last decade, possibly due to the control of some risk factors like smoking and greater access to health care. However, the mortality associated with other factors, such as obesity and diabetes, is still out of control or on the rise and is also influenced by population aging. In 2012, CVD was responsible for approximately 940,000 hospitalizations in the Brazilian Unified Health Care System (SUS) and, in relative terms, accounted for 8.3% of all causes of hospitalizations and 18.6% of costs reimbursed.

The economic impact of CVDs in public and private health care systems has been the subject of some national studies (1–3). These costs are high due to requirements from patients with CVDs of hospitalization, diagnostic procedures and revascularization, medical follow-up, and chronic use of several medications.

Familial hypercholesterolemia (FH) is a genetic disease that affects the lipoprotein metabolism with an autosomal dominant inheritance that is usually characterized by a two- to four-fold increase in blood low-density lipoprotein (LDL-c) concentration. FH is a serious disease that greatly increases the risk of premature CAD, accounting for 5-10% of coronary events occurring before the age of 50 years (4). In the absence of treatment, young FH carriers present a 90-fold increased mortality rate (5).

The diagnosis of FH is complex, and the exact prevalence of this disease is unclear in several populations, including the Brazilian one. The World Health Organization estimates about 10 million individuals with FH in the world; however, less than 10% of these have a known diagnosis of FH, and less than 25% receive lipid-lowering treatment (6).

This study aimed to estimate the impact of FH on hospitalizations due to CAD using the population attributable risk methodology and cost data from the hospital admission system of the Brazilian SUS.

## SUBJECTS AND METHODS

This was a cross-sectional and descriptive study of secondary data related to hospital reimbursement obtained from the SUS database (DATASUS). The data sources studied were the abridged files of the Hospital Information System of the SUS (SIHSUS), which controls payments for services provided by hospitals (7). Data were obtained from the standard forms for hospital admission authorization (AIH), used by public managers for prospective payment based on diagnostic-related groups (DRG), with some adaptations (8).

The number of hospitalizations is slightly greater than that of hospitalized individuals, since the same individual may be hospitalized more than once for the same reason during the considered period. Hospitalizations with a primary diagnosis (reason for hospitalization) related to CAD of possible atherosclerotic origin were identified from all hospitalizations of adults (≥ 20 years) in the SUS between 2012 and 2014. The selected international codes of diseases (ICD 10) were: I20 (angina pectoris), I21 (acute myocardial infarction), I24 (other acute ischemic heart diseases), I22 (recurrent myocardial infarction), I23 (certain complications following acute myocardial infarction), and I25 (chronic ischemic heart disease).

In order to stabilize annual fluctuations, we determined the average number of hospitalizations and hospital deaths in the triennium 2012-2014 by region of residence (North, Northeast, Southeast, South, and Central-West), sex, and age groups (20-44, 45-64, 65-74, and 75 years or older).

Costs are expressed in Brazilian reais (R$), which were converted to international dollar (Intl$) through a division by a correction factor (1.646) defined by the World Bank for the year 2013 (Purchasing Power Parity, PPP) (9).

### Attributable fraction due to familial hypercholesterolemia

The attributable risk methodology has been widely used to overcome the problem generated by the absence or underestimation of information regarding the diagnosis to be studied. This method is based on the relative risk or odds ratio for a medical condition (hospitalization for CAD) according to the presence or absence of a particular health condition (FH) and combines risk or reason with estimates of the proportion of the population with the health condition (FH prevalence) to calculate an etiological fraction. The etiologic fraction calculated in this study estimated the proportion of hospitalizations for CAD that would be attributable to the presence of FH.

For calculation of the attributable fraction of the target group by sex and age group, the following formula was used (10):


RAPi=[P×(RRi−1)]/[P×(RRi−1)+1]

where RAP_i_ is the population attributable risk fraction for the medical condition “i” due to hypercholesterolemia, P represents the prevalence of FH, and RRi is the relative risk or odds ratio of the medical condition “i” for individuals with FH compared with those without the disease.

### Prevalence of familial hypercholesterolemia and risk of coronary events

In the absence of national data on the prevalence of FH, we conducted a literature search and selected two population-based studies that estimated the prevalence of FH in the United States (unselected population of 36,949 individuals and prevalence of 0.4%, 95% confidence interval [95%CI] 0.32-0.48%) (11) and Denmark (unselected population of 69,106 individuals and prevalence of 0.73%) using the same diagnostic criteria (Dutch Lipid Clinic Network) (12,13). Considering that the prevalence of FH in Brazil is unknown and there is great racial and ethnic variability in the country, we opted to carry out the analyses based on the prevalence rates found in those two populations.

The source of relative risk estimation was obtained from a Danish general population study (The Copenhagen General Population Study) (14). In this study, the odds ratio adjusted for other risk factors for coronary events was 13.2 (95%CI 10.0-17.4) for the group with a definitive or probable diagnosis of FH. For use in the attributable fraction formula, this value was converted into a relative risk of 8.56 (14).

## RESULTS

Tables 1, 2, and 3 show the total number of hospitalizations for CAD and related costs in Brazil by sex, age group, and region of residence. Most hospitalizations (48%) occurred due to a diagnosis of angina (I20) in the age group between 45-74 years (76.3%) and in the Southeast region (47.8%). Men had more hospitalizations for all diagnosis of CAD than women (60.4% *vs*. 40.6%).

**Table 1:** Hospitalizations due to coronary artery disease attributable to familial hypercholesterolemia in the population aged ≥ 20 years distributed by sex. Annual average values for the period of 2012-2014 (Brazilian Unified Health Care System, SUS)

Coronary artery disease ICD-10	Hospitalizations		Cost per year (R$/Intl$)*		Cost per case (R$/Intl$)	
	Men	Women	Men	Women	Men	Women
I20 Angina pectoris	68,745	49,863	294,160,412	171,880,995	4,278,99	3,447,04
I21 ST elevation and non-ST elevation myocardial infarction	54,787	31,576	189,979,637	100,366,337	3,467,60	3,178,53
I22 Subsequent ST elevation and non-ST elevation myocardial infarction	13,431	8,854	69,601,281	38,064,261	5,182,27	4,298,94
I23 Certain current complications following ST elevation and non-ST elevation myocardial infarction (within the 28 day period)	1,280	752	3,945,129	2,219,376	3,081,33	2,949,99
I24 Other acute ischemic heart diseases	599	375	2,129,970	1,245,642	3,557,86	3,324,67
I25 Chronic ischemic heart disease	9,865	5,853	72,816,065	39,509,958	7,381,00	6,750,76
Total	148,707	97,274	632,632,494 / 384,345,379	353,286,570 / 214,633,396	4,254 / 2,584	3,631 / 2,206
Attributable to FH (prevalence 0.4%)	4,382	2,867	18,642,695 / 11,326,060	10,410,805 / 6,324,912	-	-
(%) of total	2.9	2.9	2.9	2.9	-	-
Attributable to FH (prevalence 0.73%)	7,808	5,107	33,215,043 / 20,179,467	18,548,772 / 11,268,999	-	-
(%) of total	5.3	5.3	5.3	5.3	-	-

ICD-10: International Classification of Diseases, Tenth Revision; R$: Brazilian Real; Intl$: International Dollar; FH: Familial Hypercholesterolemia.

* International dollar: PPP 2013; correction factor 1.646.

**Table 2:** Hospitalizations due to coronary artery disease attributable to familial hypercholesterolemia and distributed by age group. Annual average values for the period of 2012-2014 (Brazilian Unified Health Care System, SUS)

ICD-10: I20 – I25	Age groups (years)				Total
	20-44	45-64	65-74	75+	
Total CAD	18,012	123,306	64,426	40,237	245,981
COSTS (R$/Intl$)*	48,956,113 / 29,742,474	508,160,232 / 308,724,321	286,604,042 / 174,121,532	142,198,678 / 86,390,448	985,919,064 / 598,978,775
Attributable to FH (prevalence 0.4%)	531	3,634	1,899	1,186	7,249
Coef/10,000/year	0.1	0.9	2.1	2.1	0.5
COSTS (R$/Intl$)*	1,442,660 / 876,464	14,974,691 / 9,097,625	8,445,775 / 5,131,091	4,190,374 / 2,545,792	29,053,500 / 17,650,972
Attributable to FH (prevalence 0.73%)	946	6,474	3,383	2,113	12,915
Coef/10,000/year	0.1	1.6	3.7	3.7	1.0
COSTS (R$/Intl$)*	2,570,366 / 1,561,583	26,680,177 / 16,209,099	15,047,708 / 9,141,985	7,465,924 / 4,535,799	51,764,175 / 31,448,466

ICD-10: International Classification of Diseases, Tenth Revision; CAD: Coronary Artery Disease; R$: Brazilian Real; Intl$: International Dollar; FH: Familial Hypercholesterolemia; Coef/10,000/year: coefficient per 10,000 inhabitants per year.

* International dollar: PPP 2013; correction factor 1.646.

**Table 3:** Hospitalizations for coronary artery disease attributable to familial hypercholesterolemia in the population aged ≥ 20 years distributed by region of residence. Annual average values for the period of 2012-2014 (Brazilian Unified Health Care System, SUS)

ICD-10: I20 – I25	Brazilian Region					Total
	North	Northeast	Southeast	South	Central-West	
Total CAD	7,622	40,901	117,553	63,765	16,140	245,981
Coef/10,000/year	0.9	10.2	128.9	110.8	1.2	18.2
COSTS (R$/Intl$)**	25,708,081 / 15,618,518	151,315,900 / 91,929,465	475,074,666 / 288,623,733	271,105,593 / 164,705,706	62,714,824 / 38,101,351	985,919,064 / 598,978,775
Attributable to FH (prevalence 0.4%)	225	1,205	3,464	1,879	476	7,249
Coef/10,000/year*	0.2	0.3	0.6	0.9	0.5	0.5
COSTS (R$/Intl$)**	757,577 / 460,253	4,459,044 / 2,709,018	13,999,711 / 8,505,292	7,989,060 / 4,853,621	1,848,108 / 1,122,788	29,053,500 / 17,650,972
Attributable to FH (prevalence 0.73%)	400	2,147	6,172	3,348	847	12,915
Coef/10,000/year*	0.4	0.6	1.0	1.7	0.8	1.0
COSTS (R$/Intl$)**	1,349,764 / 820,026	7,944,610 / 4,826,616	24,943,070 / 15,153,749	14,233,985 / 8,647,622	3,292,746 / 2,000,453	51,764,175 / 31,448,466

ICD-10: International Classification of Diseases, Tenth Revision; CAD: Coronary Artery Disease; R$: Brazilian Real; Intl$: International Dollar; FH: Familial Hypercholesterolemia; Coef/10,000/year: coefficient per 10,000 inhabitants per year.

* Not adjusted to age.

** International dollar: PPP 2013; correction factor 1.646.

Of the total of 246,981 hospitalizations per year in individuals aged 20 years or older, 7,249 to 12,915 would be attributable to FH, assuming a prevalence of 0.4% or 0.73%, respectively. Of the total costs, 2.9-5.3% would be attributable to FH. The average cost per event was R$ 4,008 (R$ 4,254 for men and R$ 3,631 for women).

## DISCUSSION

The present study analyzed hospitalizations in the Brazilian public health system for CAD and estimated the attributable fraction of FH in these hospitalizations. CAD was responsible for approximately 2.2% of all admissions to the public health system, with an amount paid for 245,981 hospitalizations of R$ 986 million per year (Intl$ 599 million), which is equivalent to 10.6% of all public hospitalization expenses in adults (approximately R$ 9,3 billion). Considering the prevalence of FH in American and European studies, we estimated that 2.9 to 5.3% of these hospitalizations and CAD expenses would be attributable to FH (R$ 29 to 51 million/year). As expected, we observed a higher prevalence of events and costs in males, between the ages of 45-64 years, and in the Southeast region. The largest share of costs was for the treatment of angina pectoris and acute myocardial infarction, although the cost per case was higher for individuals with recurrent myocardial infarction and chronic ischemic disease.

Azambuja and cols. estimated the number of cases of severe cardiovascular disease (coronary heart disease, cerebrovascular disease, heart failure, and others) from the inpatient lethality and mortality rates and sick leaves and retirement data. Approximately 2 million cases of severe CVD were reported in 2004 in Brazil, accounting for 5.2% of the population over the age of 35 years. The annual cost corresponded to 1.74% of the national gross domestic product (GDP) in 2004 and a potential loss of 0.97% of the GDP attributed to loss of productivity (15). Ribeiro and cols. demonstrated that hospitalizations for heart failure and coronary artery revascularization (angioplasty and surgeries) were responsible for the largest share of costs in the public health system in 2012 (2). Although the number of hospitalizations due to heart failure decreased between 2007 and 2012, there was a significant increase in the number of angioplasty procedures during the same period (1).

Some studies on costs related exclusively to CAD have been conducted in Brazil. In 2011, Teich and Araujo estimated the costs of CAD from the perspectives of the public and private sectors, including indirect costs of productivity loss. Through an analysis of the historical series of hospitalizations for acute myocardial infarction, angioplasty, and revascularization (1998-2010) in the DATASUS database, the authors estimated the average costs per hospitalized patient to be R$ 5,236 and R$ 16,905 in the public and private health care systems, respectively (3). From the public perspective, the estimated values are higher than those shown in this study, possibly due to the inclusion of indirect costs attributed to loss of productivity associated with death or recovery time following the event until work activity is resumed. This share of costs accounted for 73% of the total costs. Using primary collected data, Ribeiro and cols. estimated the costs of CAD in a group of 147 patients attending a referral center in public and private health care systems in a state in southern Brazil. They demonstrated annual costs of outpatient treatment of R$ 1,488 in the public and R$ 2,094 in the private health care system for the year 2002 (16).

Our study has some limitations. In the absence of data on the prevalence of FH and risk of related events in our population and the fact that international data are not disaggregated by age, we used the same prevalence of FH for all age groups over 20 years, as well as the same relative risk for events. This prevented us from observing differences in the number of hospitalizations and costs between individuals in the general population and those with FH, as we had supposed by the early occurrence of events in this population.

DATASUS is a database with a substantial number of national registries and coverage that reflects the amounts reimbursed to health care units but does not necessarily take into account all the resources used. This database was initially developed for administrative-financial functions, and may not be free of coding and diagnostic errors, intentional or not. The data refer only to the hospitalizations of establishments owned or contracted by the public network (SUS), even though they represent an expressive part of the hospitalizations in the country. In addition, the unit of analysis is the hospitalization, not the patient; therefore, it does not include variables with explanatory potential such as age, regional differences, and severity of the clinical condition at the time of hospitalization. In addition, our data prevents us from evaluating the total cost attributable to FH in the entire country since we did not assess the impact of this disease on the private health care system.

Chronic diseases carry high costs for the patients, their families, and the entire society due to lost productivity attributed to absenteeism, medical leave, early retirement, and premature death. For a more comprehensive and realistic estimate of the costs of a chronic disease, it is necessary to include such indirect costs, which was not done in the present analysis. In addition, the outpatient costs associated with pharmacological treatment, rehabilitation, medical examinations, and consultations were also not estimated, suggesting that the estimates obtained would underestimate the real economic impact of CAD in Brazil.

The number of individuals with FH in Brazil is unknown, but is probably comparable to that in other countries, in which FH is reported to affect between 1/250 and 1/500 individuals (14). Importantly, Jannes and cols. demonstrated in a national cascade screening program (Hipercol Brasil) that early CAD was present in 12% of the index and family cases affected by FH, confirmed by molecular diagnosis. The high risk of development of ischemic events in younger age groups makes early diagnosis and treatment very important in order to minimize the great clinical and economic impact (17). Due to the high costs associated with the diagnosis and treatment of FH, international economic analyses have been conducted with the objective of estimating the cost-effectiveness of cascade genetic screening (18–20) and different therapeutic approaches for health care systems (21,22). However, it is important to consider that with the end of patents for statins and ezetimibe, which are still the treatments of choice for FH in our country, the cost of lipid-lowering treatment should reduce substantially (23).

Cost estimates of hospitalizations are essential for economic evaluations and cost-effectiveness studies of several technologies for diagnosis and treatment, old or new, aimed at a more efficient management of ischemic heart disease in Brazil. Since FH is probably responsible for high costs secondary to CAD, the diagnosis and early intervention with cholesterol-lowering medications can have a strong impact in reducing the clinical and economic burden of the disease in the country.
